# Editorial: The Double Facets of Social Behavior in Drug Addiction

**DOI:** 10.3389/fnbeh.2022.907327

**Published:** 2022-05-05

**Authors:** Rana El Rawas, Marco Venniro

**Affiliations:** ^1^Division of Psychiatry I, Department of Psychiatry, Psychotherapy, Psychosomatics and Medical Psychology, Medical University Innsbruck, Innsbruck, Austria; ^2^Department of Anatomy and Neurobiology, University of Maryland School of Medicine, Baltimore, MD, United States

**Keywords:** social interaction, drug addiction, stress, social support, reward

The Special Issue of Frontiers in Behavioral Neuroscience “*The Double Facets of Social Behavior in Drug Addiction*” includes reviews and research articles spanning from preclinical animal models to human studies.

Social behaviors are important determinants for the etiology of several neuropsychiatry disorders, such as substance use disorders. Social interactions as an alternative to addictive drugs, in the form of social play and social support, tend to be protective against initiation, maintenance or relapse to drug use in both laboratory animals and humans. Conversely, negative social interaction in the form of stress and peer pressure, has an opposite effect with increasing drug use and relapse. This Special Issue covers the double facets of social behaviors in modulating substance use disorders with six research articles and one review.

Pomrenze et al. reviewed the literature about the neurobiology of social brain. The authors focused on the effects of social defeat and social isolation, two major relevant social stressors, on drug dependence, on the neural circuits and the molecular mechanisms by which these stressors enhance vulnerability to drugs use. Additionally, the authors discussed how a history of drug use can also affect social behaviors, inducing a continuous circle of socio-addictive behaviors that influence each other. Finally, they identified candidate brain mechanisms that underlie the competitive nature of social and drug-rewards.

Shuai et al. investigated the mechanisms underlying the alcohol harm paradox when socioeconomic deprivation is associated with greater alcohol problems despite lower alcohol consumption. The authors evaluated a multistage causal risk pathway that has been proposed to contribute to the alcohol harm paradox, in the form of questionnaires assessed by hazardous drinkers from an undergraduate population. The authors found that socioeconomic deprivation increased the probability of being exposed to aversive experiences, which enhanced the risk of internalizing symptoms, which augmented the risk of drinking to cope, which conferred susceptibility to alcohol dependence.

Sharp and Smith determined the extent to which drugs that increase synaptic dopamine, norepinephrine, and serotonin influence the positive reinforcing effects of social contact relative to a non-social stimulus in both male and female rats. The study showed that increases in extracellular dopamine, but not extracellular norepinephrine or serotonin, enhance the positive reinforcing effects of social contact in both male and female rats without affecting the responding maintained by a non-social stimulus.

Altshuler et al. explored the effect of housing conditions on incubation of oxycodone craving in rats with a history of oxycodone self-administration during either adult or adolescent phases. The authors reported no age differences in oxycodone self-administration and incubated oxycodone seeking at late abstinence phase after forced abstinence. Critically, the authors found that group-housing decreased oxycodone craving relative to the social isolation group showing the importance of social support during abstinence.

Fulenwider et al. characterized the effects of social housing on ethanol intake in male mice using a two-bottle choice procedure. The authors reported that social-housed mice consumed more ethanol compared to single-housed mice. However, the overall intake levels of social-housed mice were lower than the levels of single-housed mice. This suggests that while environmental enrichment attenuates ethanol intake, social enrichment could potentiate it. Additionally, using immunohistochemistry, the authors reported a correlation between ethanol intake and FosB-positive cells in the Edinger-Westphal nucleus and nucleus accumbens shell in individually housed mice, but not in socially housed mice.

Seidisarouei et al. using a novel behavioral social-sucrose behavioral procedure, classified the ultrasonic vocalization subtype for both sucrose and social reward. The authors demonstrated that time spent with the social stimuli was dependent on the concentration of sucrose available. Additionally, they found a difference in the emission of flat and frequency-modulated calls in the social and non-social reward zones. Lastly the authors reported a large variability in the rat vocalization repertoire toward non-social and social rewards.

Kamens et al. investigated the biological and genetic mechanisms underlying the adolescent stress-induced long-term risk of opioid use in male and female C57BL/6J and BALB/cJ mice. The authors reported that adolescent stress did not influence morphine sensitization or consumption in BALB/cJ animals, and there was limited evidence of stress effects in female C57BL/6J mice. In contrast, male C57BL/6J mice exposed to chronic variable social stress during adolescence had blunted morphine sensitization compared to control animals. Additionally, C57BL/6J mice exposed to social stress during adolescence had attenuated corticosterone recovery following an acute stressor and downregulation of twelve miRNA in the prefrontal cortex compared to control mice.

Altogether, this Special Issue highlights the importance of incorporating different aspects of social behavior spanning from housing conditions to sensory cues and different types of stressors in the investigation of the behavioral and biological mechanisms underlying its effect on substance use disorders with different abused drugs. Finally, we hope that this collection of articles will facilitate the development of fora and discussion groups to improve translational utility of animal models together with better social-based interventions for substance use disorders.

## Author Contributions

RE and MV wrote the editorial. All authors approved the submitted version.

## Conflict of Interest

The authors declare that the research was conducted in the absence of any commercial or financial relationships that could be construed as a potential conflict of interest.

## Publisher's Note

All claims expressed in this article are solely those of the authors and do not necessarily represent those of their affiliated organizations, or those of the publisher, the editors and the reviewers. Any product that may be evaluated in this article, or claim that may be made by its manufacturer, is not guaranteed or endorsed by the publisher.

